# Real-world efficacy and safety of naltrexone-bupropion therapy in Chinese patients with obesity: A single-centre experience

**DOI:** 10.1007/s12020-024-04029-2

**Published:** 2024-10-05

**Authors:** David Tak Wai Lui, Kimberly Hang Tsoi, Carol Ho Yi Fong, Nancy Su Jiang, Wing Sun Chow, Michele Mae Ann Yuen

**Affiliations:** Department of Medicine, School of Clinical Medicine, Li Ka Shing Faculty of Medicine, The University of Hong Kong, Hong Kong, Hong Kong

**Keywords:** (MeSH): obesity, Asian, East Asian people, bupropion hydrochloride, naltrexone hydrochoride drug combination, body weight

## Abstract

**Purpose:**

Naltrexone-bupropion (Contrave®) has shown efficacy and safety in large randomised controlled trials, predominantly comprising Caucasians. Data are limited in Asian populations. We carried out a retrospective matched cohort study of Chinese patients with obesity to evaluate the efficacy and safety of naltrexone-bupropion in real-world clinical practice.

**Methods:**

We performed a retrospective matched cohort study of Chinese patients with obesity managed in the Obesity Clinic of Queen Mary Hospital in Hong Kong between 1 January 2016 and 31 December 2020. Electronic health records of patients treated with naltrexone-bupropion were retrieved for body weight and height, obesity-related metabolic parameters, and adverse events over a 12-month period. Age- and sex-matched controls from the Obesity Clinic who were only on self-directed lifestyle management were identified for comparison of weight changes. General linear models were used to analyse the change in body weight over 12 months.

**Results:**

Thirty-seven patients treated with naltrexone-bupropion were included (mean age 42.2 ± 8.4 years, 54.1% men, baseline body mass index 37.3 ± 4.6 kg/m^2^), and 37 age- and sex-matched controls were included. Among the 37 naltrexone-bupropion-treated patients, the mean weight loss was 9.2 ± 5.2% at 6 months and 9.7 ± 8.1% at 12 months, which were significantly more than in controls (p < 0.001). Improvements in the obesity-related parameters were observed in association with weight loss over 12 months. Ten patients (27.0%) discontinued naltrexone-bupropion due to side effects, mainly neurological and gastrointestinal manifestations, within the first 12 months.

**Conclusion:**

We demonstrated real-world efficacy and safety of naltrexone-bupropion among Chinese patients with obesity.

## Introduction

Obesity is a disease where an excessive accumulation of body fat leads to impaired health and an increased risk of long-term health complications and mortality [1]. Obesity is fast becoming a global pandemic. According to the World Obesity Atlas 2023, the global prevalence of obesity is expected to rise from 14% in 2020 to 24% by 2035. In recent years, there have been concerns about the rising prevalence of obesity in the Asia-Pacific regions, contributed by the adoption of Westernised lifestyles amongst other factors [2–4]. The rise is particularly alarming in countries such as China, which was once considered to have one of the leanest populations in the world. The number of Chinese people with obesity was below 0.1 million in 1975 and rose to 43.2 million in 2014, accounting for 16.3% of the global obesity burden [5].

Effective anti-obesity pharmacotherapies have emerged in the recent years, which can bring about significant weight loss and help in weight management [6]. Naltrexone-bupropion combination (Contrave®) is one of the classes of approved anti-obesity pharmacotherapies. Although the exact mechanism of action is not fully understood, it is hypothesised that bupropion stimulates hypothalamic pro-opiomelanocortin (POMC) cell, with downstream effects to reduce food intake and increase energy expenditure. On the other hand, naltrexone blocks opioid receptors and the autoinhibition of POMC cells, augmenting POMC firing in a synergistic manner [7, 8]. The efficacy and safety profile of naltrexone-bupropion has been established by several multicentre randomised double-blind placebo-controlled trials in the Contrave Obesity Research (COR) programme, including the Contrave Obesity Research-I (COR-I) study, the Contrave Obesity Research-II (COR-II) study, and the Contrave Obesity Research-Intensive Behavior Modification study (COR-BMOD) [9–11]. The use of naltrexone-bupropion was associated with 4.8% placebo-corrected weight loss over one year [6]. In addition to weight loss, the studies also demonstrated improvements in some markers of cardiometabolic risk, weight-related quality of life, and control of eating, without evidence of negative effects on depression or suicidality [9–11].

Taken together, naltrexone-bupropion is a promising option for patients with obesity who have failed lifestyle modifications. Nonetheless, recruited population in the COR programme consists mainly of Caucasians [12]. There are limited data regarding the use of naltrexone-bupropion in the Asian populations. Furthermore, it is helpful to evaluate whether the benefits of naltrexone-bupropion shown in the randomised controlled trial translate to the real-world clinical practice setting, since subjects followed up in the randomised controlled trial are often more intensively monitored and may be more motivated [13]. Hence, we carried out a retrospective cohort study of Chinese patients with obesity to evaluate the efficacy and safety of naltrexone-bupropion use.

## Materials and Methods

We performed a retrospective cohort study of patients with obesity in the Obesity Clinic of Queen Mary Hospital, Hong Kong SAR. In the Obesity Clinic, each patient with obesity was given advice on lifestyle modifications (dietary advice and advice on physical activities). Suitable patients with body mass index (BMI) above the action point of 27.5 kg/m^2^ in Asians and who were indicated for pharmacotherapy were all offered the option of using naltrexone-bupropion. Patients who agreed to start naltrexone-bupropion were prescribed the medication after evaluation for contraindications [14, 15]. Patients started on naltrexone-bupropion were scheduled to return for follow-up visits at 1 month, 2 months, 3 months, 6 months, and every 6 monthly thereafter. The study included dose escalation as recommended by the manufacturer, beginning with a quarter of the full dose (naltrexone 8 mg/bupropion 90 mg once daily) at week 1 and gradual increment weekly to the full dose (naltrexone 32 mg/bupropion 360 mg in 2 divided doses) at week 4. The initial follow-up visits at 1 month and 2 months were mainly for review of drug tolerance. Body height was measured at baseline and body weight was measured at each clinic visit. Blood was taken after an overnight fast for at least 8 h at baseline, 3 months and at the subsequent visits. Biochemical parameters measured included glycated haemoglobin (HbA1c), lipid profile, alanine aminotransferase (ALT), and aspartate aminotransferase (AST). Patients were invited to undergo vibration-controlled transient elastography (VCTE) at baseline and 12 months for assessment of the levels of hepatic steatosis and fibrosis as determined by controlled attenuation parameter (CAP) and liver stiffness (LS) measurements. The methods for performing VCTE have been described in our previous publication [16].

In this study, we included Chinese patients with BMI ≥ 27.5 kg/m^2^ who were started on naltrexone-bupropion between 1 January 2016 and 31 December 2020. To evaluate the effect of naltrexone-bupropion on body weight, an age- and sex-matched control group consisting of patients with obesity who were indicated for pharmacotherapy but only on self-directed lifestyle management in the same Obesity Clinic of Queen Mary Hospital were identified during the same inclusion period. The study followed the principles in the Declaration of Helsinki and was approved by the Institutional Review Board of the University of Hong Kong/Hospital Authority Hong Kong West Cluster (IRB Ref.: UW 15-126).

### Definition of covariates

Dyslipidemia was defined by fasting triglycerides (TG) ≥ 1.69 mmol/L, high-density lipoprotein cholesterol (HDL-C) < 1.04 mmol/L in men and <1.29 mmol/L in women, and low-density lipoprotein cholesterol (LDL-C) ≥ 3.4 mmol/L, or on lipid-lowering agents. Hypertension was defined by blood pressure ≥140/90 mmHg or on anti-hypertensive medications. Diabetes was defined by fasting glucose ≥7.0 mmol/L, HbA1c ≥ 6.5%, or the use of anti-diabetic medications.

### Outcomes

The primary outcome was the percentage of body weight loss associated with naltrexone-bupropion over 12 months, in comparison with body weight loss in the control group. In addition, we reported the percentage of excess body weight loss (%EBWL). The %EBWL is calculated using an ideal body weight of BMI 23 kg/m^2^ with the formula: %EBWL = (baseline weight – follow-up weight)/(baseline weight – ideal body weight) x 100 [17].

There were several secondary outcomes:

(i) The proportion of individuals achieving ≥5% or ≥10% weight loss compared to baseline at 6 months and 12 months;
(ii) Changes in the obesity-related laboratory parameters at 3 months, 6 months, and 12 months;
(iii) Percentage body weight loss among patients treated with naltrexone-bupropion beyond 12 months; and
(iv) Side effects leading to termination of naltrexone-bupropion.

### Statistical analyses

All statistical analyses were performed with IBM^®^ SPSS^®^ Statistics version 26 for Windows. Two-sided p-values < 0.05 were considered statistically significant. Results were reported as means ± standard deviations, medians with interquartile ranges (IQR) for skewed data, or percentages as appropriate. Between-group comparisons of clinical and laboratory parameters were performed with t-test and Chi-square test or Fisher’s exact test as appropriate. Generalised mixed models with repeated measures were used to examine mean changes in clinical and laboratory parameters at baseline, 3 months, 6 months, and 12 months.

The analyses of efficacy were performed based on all included patients with a baseline weight and ≥1 post-baseline weight on naltrexone-bupropion. Missing data were imputed by carrying forward the last observation while on naltrexone-bupropion.

### Sample size calculation

The primary outcome was the percentage of body weight loss associated with naltrexone-bupropion over 12 months. A sample size of 11 in the naltrexone-bupropion group would be necessary to attain an 80% power and a 5% significance level for detecting a mean percent change of body loss of 5% from baseline, assuming the standard deviation of the differences being 5%.

## Results

We included 37 patients who were started on naltrexone-bupropion and 37 age- and sex-matched controls. The two groups were comparable in terms of baseline characteristics (Table 1).

**Table 1:** Baseline characteristics of the naltrexone-bupropion and the control groups

	Naltrexone-bupropion (*n* = 37)	Control (*n* = 37)
Age (years)	42.2 ± 8.4	41.9 ± 10.1
Male (%)	20 (54.1%)	20 (54.1%)
Chinese	37 (100%)	37 (100%)
Body weight (kg)	103.7 ± 18.2	97.2 ± 13.1
Body mass index (kg/m^2^)	37.3 ± 4.6	35.6 ± 4.1
Waist circumference (cm)	110 ± 9.9	N/A
Waist-hip ratio	0.92 ± 0.07	N/A
Hypertension (%)	32 (86.5%)	27 (73.0%)
Diabetes (%)	16 (43.2%)	9 (24.3%)
Dyslipidaemia (%)	26 (70.3%)	28 (75.7%)
Obstructive sleep apnoea (%)	22 (59.4%)	23 (62.2%)
Previous bariatric surgery (%)	4 (10.8%)	0 (0%)

*N/A* not available

Data were presented as mean ± standard deviation or numbers (%) as appropriate

**Fig. 1:**
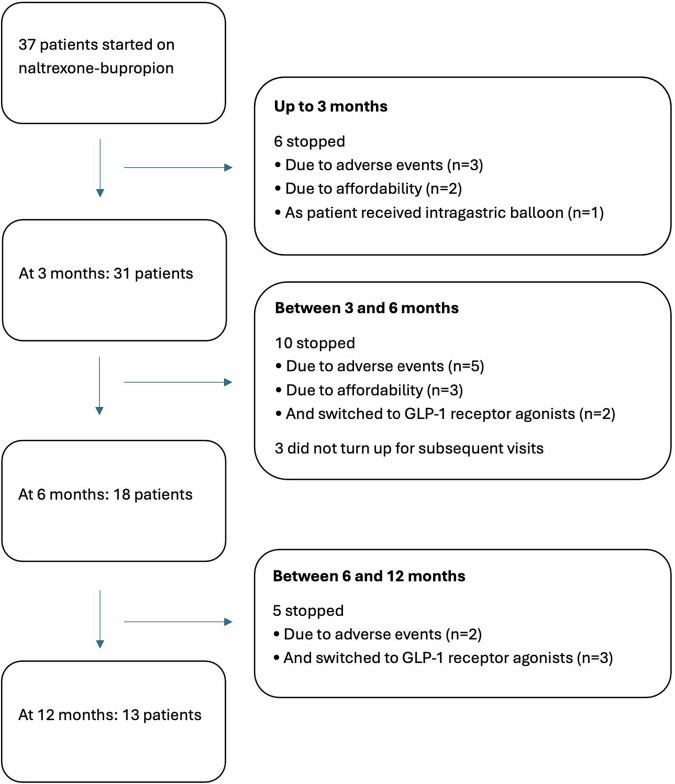
Study flow diagram

**Fig. 2:**
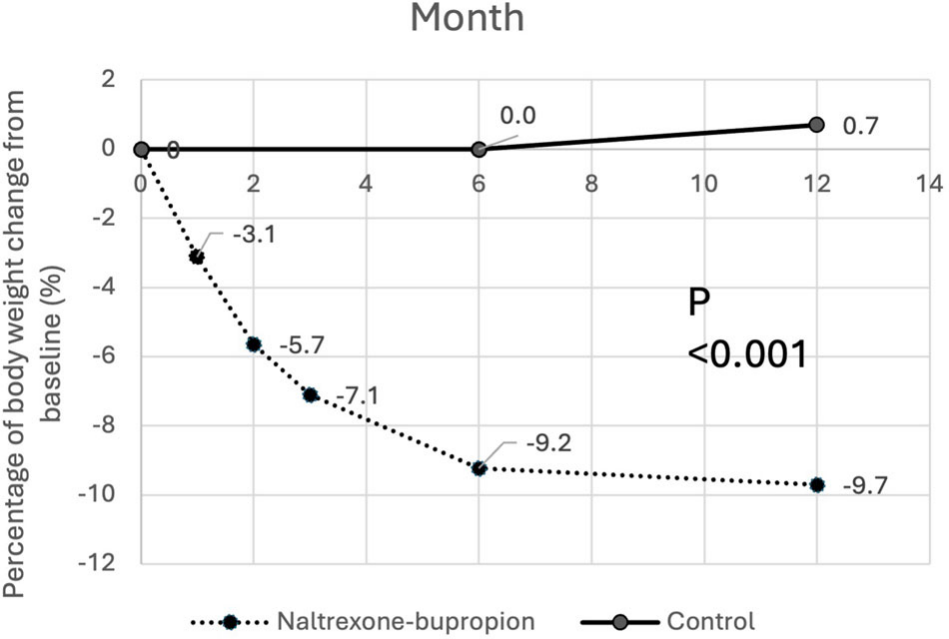
Percentage of body weight change from baseline over the 12 months of follow-up. Naltrexone-bupropion treatment was associated with clinically significant weight loss of close to 10%, reaching a nadir at 6 months and sustaining through 12 months

Patients treated with naltrexone-bupropion were followed up for a median of 7.2 months (IQR: 3.2–25.9). Figure 1 is the study flow diagram. Among the 37 patients who were started on naltrexone-bupropion, 4 patients discontinued treatment within one month and hence were excluded from the subsequent analysis. Eighteen patients (48.6%) were followed up till 6 months, and 13 patients (35.1%) were followed up till 12 months. Twenty-three patients (62.6%) reached the standard dose, 6 (16.2%) reached a dose of 3 tablets per day, and 4 (10.8%) reached a dose of 2 tablets per day. Naltrexone-bupropion treatment was associated with clinically significant weight loss of close to 10%, reaching a nadir at 6 months and sustaining through 12 months (Table 2; Fig. 2). When expressed in %EBWL, naltrexone-bupropion treatment group achieved a mean of around 25% at 6 months and 28% at 12 months (Table 2). In contrast, there was no significant weight change in the control group. The body weight loss achieved by the naltrexone-bupropion group was statistically significantly more than that in the control group (all p < 0.001), confirming with its efficacy.

**Table 2:** Change in body weight at 6 months and 12 months in the naltrexone-bupropion treatment and control group

	6 months			12 months		
Measure	Naltrexone-bupropion	Control	p-value	Naltrexone-bupropion	Control	p-value
Number of participants						
LOCF	18	35	–	13	35	–
Completers	15	29	–	13	28	–
ΔBody weight, kg						
LOCF	−9.8 ± 5.4	−0.1 ± 3.4	**<0.001**	−10.1 ± 8.4	0.7 ± 4.0	**<0.001**
Completers	−10.6 ± 5.6	0.3 ± 3.6	**<0.001**	−10.1 ± 8.4	1.6 ± 3.5	**<0.001**
ΔBody weight, %						
LOCF	−9.2 ± 5.2	−0.04 ± 3.3	**<0.001**	−9.7 ± 8.1	0.7 ± 4.0	**<0.001**
Completers	−9.8 ± 5.5	0.3 ± 3.5	**<0.001**	−9.7 ± 8.1	1.6 ± 3.5	**<0.001**
EBWL, %						
LOCF	25.3 ± 16.7	−0.2 ± 9.9	**<0.001**	28.0 ± 24.2	−2.5 ± 11.5	**<0.001**
Completers	27.5 ± 17.6	−1.2 ± 10.6	**<0.001**	28.0 ± 24.2	−5.0 ± 10.0	**<0.001**
Participants with ≥5% weight loss						
LOCF	16 (88.9%)	2 (5.7%)	**<0.001**	10 (76.9%)	2 (5.7%)	**<0.001**
Completers	14 (93.3%)	1 (3.4%)	**<0.001**	10 (76.9%)	1 (3.6%)	**<0.001**
Participants with ≥10% weight loss						
LOCF	5 (27.8%)	1 (2.9%)	**<0.001**	6 (46.2%)	1 (2.9%)	**<0.001**
Completers	5 (33.3%)	1 (3.4%)	**<0.001**	6 (46.2%)	0 (0.0%)	**<0.001**

*LOCF* last observation carried forward, *EBWL* excess body weight loss. Data were presented as mean ± standard deviation or numbers (%)

Bold values indicate statistical significance.

We then evaluated the proportion of naltrexone-bupropion-treated patients who achieved significant weight loss. At 6 months, around 90% of the patients achieved ≥5.0% weight loss and around 30% of the patients achieved ≥10% weight loss. At 12 months, 76.9% of the patients achieved ≥5.0% weight loss and 46.2% of the patients achieved ≥10% weight loss. In contrast, in the control group, only a minority of them achieved clinically significant weight loss (p < 0.001 in all comparisons). (Supplementary Table 1).

Table 3 summarises the changes in the obesity-related parameters among the naltrexone-bupropion-treated patients. We observed statistically significant, though modest, improvements in several obesity-related parameters: HbA1c, LDL-C, ALT, and AST levels. On the other hand, there was no significant change in blood pressure, pulse rate, HDL-C and triglyceride levels. Among the 13 patients who had complete data for Controlled Attenuated Parameter (CAP) and Liver Stiffness (LS) by Fibroscan, CAP significantly decreased from baseline of 339 dB/m (IQR: 301–400) to 299 dB/m (IQR: 244–345) at 12 months (p = 0.008), whereas LS decreased from baseline of 6.2 kPa (IQR: 5.0–9.9) to 5.7 kPa (IQR: 4.1–6.3) at 12 months (p = 0.013).

**Table 3:** Comparison of laboratory parameters at baseline, 3 months, 6 months, and 12 months

Laboratory parameters	Baseline	3 months	6 months	12 months	P value
Number of participants					
LOCF	37	22	18	13	–
Completers	37	20	15	13	–
SBP (mmHg)					
LOCF	136 ± 18	136 ± 14	128 ± 14	129 ± 16	0.135
Completers	136 ± 18	135 ± 14	128 ± 16	128 ± 16	0.098
DBP (mmHg)					
LOCF	81 ± 12	82 ± 12	82 ± 10	82 ± 9	0.793
Completers	81 ± 12	82 ± 9	82 ± 10	82 ± 9	0.919
Pulse (per minute)					
LOCF	85 ± 14	84 ± 14	80 ± 14	80 ± 17	0.415
Completers	85 ± 14	85 ± 15	78 ± 14	82 ± 16	0.449
HbA1c (%)					
LOCF	5.9 ± 1.0	5.9 ± 1.0	5.8 ± 1.0	5.7 ± 1.0	**0.044**
Completers	5.9 ± 1.0	5.9 ± 1.1	5.9 ± 1.2	5.8 ± 1.1	**0.048**
HDL-C (mmol/L)					
LOCF	1.2 ± 0.3	1.2 ± 0.2	1.2 ± 0.3	1.3 ± 0.2	0.249
Completers	1.2 ± 0.3	1.2 ± 0.2	1.2 ± 0.3	1.3 ± 0.2	0.304
LDL-C (mmol/L)					
LOCF	2.7 ± 0.7	2.5 ± 0.7	2.5 ± 0.9	2.3 ± 0.6	**0.048**
Completers	2.7 ± 0.7	2.4 ± 0.7	2.5 ± 1.0	2.4 ± 0.7	0.081
Triglycerides^a^ (mmol/L)					
LOCF	1.2 (0.9–1.9)	1.1 (0.9–1.8)	1.1 (0.9–2.1)	1.3 (0.8–1.7)	0.086
Completers	1.2 (0.9–1.9)	1.1 (0.9–1.6)	1.6 (0.9–2.2)	1.3 (0.9–1.5)	0.131
ALT^a^ (U/L)					
LOCF	28 (22–39)	30 (22–39)	25 (21–36)	23 (18–26)	**0.024**
Completers	28 (22–39)	30 (21–40)	24 (21–30)	23 (17–26)	**0.033**
AST^a^ (U/L)					
LOCF	23 (18–27)	22 (19–30)	20 (18–28)	20 (14–23)	**0.017**
Completers	23 (18–27)	22 (19–31)	20 (18–23)	17 (14–23)	**0.018**

^a^Log-transformed before analysis. LOCF, the last observation carried forward

Data were presented as mean ± standard deviation or median (interquartile range) as appropriate

Bold values indicate statistical significance.

Among the 37 patients initially put on naltrexone-bupropion, 11 of them (29.7%) continued naltrexone-bupropion up to 18 months, 9 of them (24.3%) continued up to 24 months and 8 of them (21.6%) continued up to 30 months. For the patients who persisted on naltrexone-bupropion treatment, they were able to achieve a mean of 10% body weight loss (Supplementary Table 2).

A total of ten patients (27.0%) discontinued naltrexone-bupropion due to side effects, all within the first 12 months. Among the four patients who discontinued naltrexone-bupropion early within the first month, three of them discontinued due to side effects: one for disturbances in attention and palpitations, one for neurological symptoms (headache, dizziness, and vomiting), and one discontinued due to gastrointestinal side effects of bloating. By 3 months, three patients discontinued for gastrointestinal side effects, tremor, and irritability respectively. By 6 months, two more patients discontinued for insomnia. By 12 months, two more patients discontinued for lower urinary tract symptoms and difficult-to-control hypertension respectively (Supplementary Table 3).

## Discussion

Our study reported the real-world efficacy and safety of naltrexone-bupropion among Chinese patients with obesity, which was consistent with the findings from the COR programme comprising mainly Caucasian populations. Our study demonstrated a mean of 10% body weight loss achieved by 6 months which was maintained over a 12-month follow-up period. There were also concomitant improvements in obesity-related parameters. Naltrexone-bupropion was tolerated by most of the patients in the cohort, although some did discontinue the drug due to side effects such as neurological and gastrointestinal manifestations.

Our study reported a mean of 10% weight loss among the naltrexone-bupropion users, which was more than that seen in the COR programme. The body weight and BMI of our cohort were largely similar to those seen in the COR programme. Several reasons may explain the higher percentage of body weight loss in our cohort. Firstly, as our cohort consists of exclusively Chinese individuals, it is possible that Asians may be more responsive to a similar dosage of naltrexone-bupropion compared with the Caucasians. Indeed, 27% of the cohort was treated with a lower than maximum dose of naltrexone-bupropion. This is similarly seen for glucagon-like peptide-1 receptor agonist such as liraglutide where two studies have reported clinically relevant weight loss achieved at lower doses of liraglutide in Asian populations [18, 19]. Secondly, naltrexone-bupropion is not reimbursed in Hong Kong. That means patients have to pay for the medication. Hence, the naltrexone-bupropion users could be more motivated to achieve weight loss in combination with lifestyle modifications. Consistent with the percentage body weight loss achieved among the naltrexone-bupropion-treated patients in our cohort, we observed that close to 80% of the naltrexone-bupropion-treated patients achieved ≥5% weight loss. This rate was higher than that observed in COR programme of around 50–60% [20].

It has been shown that 5–10% total body weight loss is associated with a reduction in the risk of various metabolic, skeletal, and anatomical complications of obesity [21]. Consistent with the mean of 10% weight loss observed in the naltrexone-bupropion group, which was maintained through 12 months, we observed modest improvements in the obesity-related parameters in several domains relevant to cardiometabolic risks: glycaemic control (HbA1c), lipid metabolism (LDL-C levels), liver parameters (ALT and AST levels, CAP and LS on VCTE). While improvements in glycaemic control and lipid metabolism have been reported, we have extended the observation to show that naltrexone-bupropion treatment can bring about modest improvements in liver-related parameters, likely through the improvement in insulin resistance from the weight loss. This suggests potential beneficial effects of naltrexone-bupropion on non-alcoholic fatty liver disease which commonly affects patients with obesity. Though the changes in the cardiometabolic risk factors as defined by blood-related parameters were rather modest in our study, it would be interesting to observe whether the magnitude of such changes would be bigger in a cohort with more advance-staged obesity-related comorbidities with higher initial HbA1c, ALT and AST levels. In addition, it would be interesting to observe whether a more significant improvement in hepatic steatosis would be possible over a longer treatment duration.

Two randomised controlled trials have evaluated the use of naltrexone-bupropion beyond 1 year: IGNITE [22] and LIGHT [23]. Participants of the IGNITE trial were observed up to 78 weeks, demonstrating maintenance of weight loss achieved at 26 weeks [22]. In the LIGHT trial which aimed to address the cardiovascular safety of naltrexone-bupropion, participants were followed up till 104 weeks. Results of the LIGHT trial showed that the weight loss achieved at 26 weeks was maintained through 104 weeks [23]. Our results generated from real-world clinical practice aligned with the observations from the above two trials. We observed that patients who were on naltrexone-bupropion were able to maintain the weight loss achieved at 6 months up till 30 months, showing the real-world effectiveness of naltrexone-bupropion in the longer term.

We observed a drop-out rate of 64.9% at 12 months among the naltrexone-bupropion-treated patients. This was higher than the drop-out rates observed in the trials within the COR programme, which were around 40–50% [20]. The higher drop-out rate in our cohort could be related to the costs associated with naltrexone-bupropion. The patients in this study were followed-up in a government-funded hospital, where the usual cost of care is a nominal co-payment of approximately $2 USD per drug prescribed. As naltrexone-bupropion was an unregistered drug at the time of this study and required the use of multiple third-party distributors to import under a named-patient program, the cost of this drug was close to 300-fold that of the usual drug cost for the patients. As such, the threshold for treatment termination was low and patients were keen to stop the drug with any discomfort, especially in the maintenance phase of treatment.

Regarding the safety profile of naltrexone-bupropion, our experience was in line with those observed in the COR programme [20]. The most common side effects were also gastrointestinal and neurological in nature. In our cohort, 27% of the cohort discontinued naltrexone-bupropion due to side effects. This was higher than that observed in the randomised controlled trial such as the COR-I trial which reported a rate of 19.5% [9]. This reflects the difference in the real-world clinical practice where patients who experienced side effects might more easily decide to stop the therapy as they perceived benefits from naltrexone-bupropion outweighed by additional considerations such as cost. Importantly and reassuringly, we did not observe in our cohort serious adverse events such as suicidal thoughts or seizures.

Our study is the first to show the efficacy and safety of naltrexone-bupropion among Asians, which seems to be comparable to those seen in the COR studies, which comprise of predominantly Caucasians subjects. There are several limitations to our study. Firstly, naltrexone-bupropion was very expensive in Hong Kong at the time of this study. There is a selection bias for highly motivated patients who are willing to pay to be included in the treatment cohort. Confounding by indication is therefore a potential bias of the study, which might exaggerate the weight-loss efficacy of naltrexone-bupropion. Secondly, this was a single-centre study with a relatively small sample size. However, as this is the first experience of the use of naltrexone-bupropion among Asians, we felt that the results are still important in providing insights on how this drug combination works in this cohort of patients. Finally, our cohort was 100% Chinese and our findings may not be generalizable to other Asians. Further studies in other parts of Asia will validate our findings.

## Conclusion

In this single-centre retrospective cohort study of Chinese patients with obesity treated with naltrexone-bupropion, we observed a mean of 10% body weight loss achieved by 6 months, which was maintained over the 12 months of follow-up. This was associated with improvement in obesity-related parameters. Naltrexone-bupropion was tolerated by most of the patients in the cohort, although a minority of them discontinued due to side effects such as neurological and gastrointestinal manifestations. We demonstrated the usefulness of naltrexone-bupropion in real-world clinical practice among patients with obesity.

## Supplementary information

Supplementary Tables1-3
