# Supplementary material for: Real-world efficacy and safety of naltrexone-bupropion therapy in Chinese patients with obesity: A single-centre experience
Source: Endocrine. 2024 Oct 5;87(2):522–9. doi: 10.1007/s12020-024-04029-2 (PMC11811244; doi:10.1007/s12020-024-04029-2)
Supplement: Supplementary file 1 — Supplementary Tables1-3 [file 12020_2024_4029_MOESM1_ESM.docx]

**Supplementary Table 1**. Change in body weight in the naltrexone-bupropion group over 12 months

| Measure | 2 months | 3 months | 6 months | 12 months | p-value |
| --- | --- | --- | --- | --- | --- |
| Number of participants |  |  |  |  |  |
| LOCF | 26 | 22 | 18 | 13 | -- |
| Completers | 23 | 20 | 15 | 13 | -- |
| ΔBody weight, kg |  |  |  |  |  |
| LOCF | -5.2±4.0 | -7.7±4.8 | -9.8±5.4 | -10.1±8.4 | <0.001 |
| Completers | -5.2±3.8 | -7.7±5.0 | -10.6±5.6 | -10.1±8.4 | <0.001 |
| ΔBody weight, % |  |  |  |  |  |
| LOCF | -4.8±3.8 | -7.2±4.7 | -9.2±5.2 | -9.7±8.1 | <0.001 |
| Completers | -4.7±3.5 | -7.3±4.8 | -9.8±5.5 | -9.7±8.1 | <0.001 |
| EBWL, % |  |  |  |  |  |
| LOCF | 12.7±10.6 | 19.8±14.7 | 25.3±16.7 | 28.0±24.2 | <0.001 |
| Completers | 12.1±9.7 | 20.3±15.3 | 27.5±17.6 | 28.0±24.2 | <0.001 |
| Participants with ≥5% weight loss |  |  |  |  |  |
| LOCF | 10 (38.5%) | 15 (68.2%) | 16 (88.9%) | 10 (76.9%) | <0.001 |
| Completers | 9 (39.1%) | 14 (70.0%) | 14 (93.3%) | 10 (76.9%) | <0.001 |
| Participants with ≥10% weight loss |  |  |  |  |  |
| LOCF | 4 (15.2%) | 5 (22.7%) | 5 (27.8%) | 6 (46.2%) | 0.025 |
| Completers | 3 (13.0%) | 5 (25.0%) | 5 (33.3%) | 6 (46.2%) | 0.038 |

LOCF, last observation carried forward; EBWL, excess body weight loss.

**Supplementary Table 2**. Change in body weight beyond 12 months among naltrexone-bupropion group

| Measure | 12 months | 18 months | 24 months | 30 months |
| --- | --- | --- | --- | --- |
| Number of completers | 13 | 11 | 9 | 8 |
| ΔBody weight, kg | -10.1±8.4 | -10.8±9.3 | -11.2±10.3 | -11.1±9.1 |
| ΔBody weight, % | -9.7±8.1 | -10.0±8.7 | -10.7±9.8 | -10.5±8.8 |
| EBWL, % | 28.0±24.2 | 28.6±25.7 | 31.0±27.9 | 29.8±24.8 |
| Participants with ≥5% weight loss | 10 (76.9%) | 10 (90.9%) | 7 (77.8%) | 7 (87.5%) |
| Participants with ≥10% weight loss | 6 (46.2%) | 3 (27.3%) | 3 (33.3%) | 2 (25.0%) |

LOCF, last observation carried forward; EBWL, excess body weight loss. Data were presented as mean ± standard deviation or numbers (%) as appropriate

**Supplementary Table 3.** Reasons for stopping naltrexone-bupropion due to adverse effects

| **Patient no.** | **Adverse effects** | **Duration of use (months)** | **Dosage achieved (1-4 tablets)*** |
| --- | --- | --- | --- |
| 1 | Disturbances in attention, palpitations | 1 | 3 |
| 2 | Headache, dizziness and vomiting | 1 | 3 |
| 3 | Bloating | 1 | 4 |
| 4 | Gastrointestinal side effects | 3 | 2 |
| 5 | Tremor | 3 | 3 |
| 6 | Irritability | 3 | 2 |
| 7 | Insomnia | 6 | 4 |
| 8 | Insomnia | 6 | 2 |
| 9 | Lower urinary tract symptoms | 10 | 4 |
| 10 | Difficult-to-control hypertension | 12 | 4 |

*One tablet contains naltrexone 8mg/bupropion 90mg
